# Inequalities in early initiation of breastfeeding in Bangladesh: an estimation of relative and absolute measures of inequality

**DOI:** 10.1186/s13006-023-00584-y

**Published:** 2023-08-28

**Authors:** Satyajit Kundu, Syed Sharaf Ahmed Chowdhury, Md Tamzid Hasan, Azaz Bin Sharif

**Affiliations:** 1 Global Health Institute, North South University, Dhaka, 1229 Bangladesh; 2 Faculty of Nutrition and Food Science, Patuakhali Science and Technology University, Patuakhali, 8602 Bangladesh; 3 Department of Public Health, North South University, Dhaka, 1229 Bangladesh

**Keywords:** Early initiation of breastfeeding, Timely initiation of breastfeeding, Inequalities, Disparities, Bangladesh, BDHS

## Abstract

**Background:**

Evidence suggested that inequalities based on education, wealth status, place of residence, and geographical regions significantly influence the key breastfeeding indicators including early initiation of breastfeeding. This study aimed to estimate the trends and magnitude of inequalities in early initiation of breastfeeding practice in Bangladesh from 2004 to 2017 applying both absolute and relative measures of inequality.

**Methods:**

We used data from the last five Bangladesh Demographic Health Survey (BDHS) from 2004 to 2017 to measure the inequalities in early initiation of breastfeeding practice using the WHO’s Health Equity Assessment Toolkit (HEAT) software. Following summary measures were estimated to measure the inequalities: Population Attributable Risk (PAR), Population Attributable Fraction (PAF), Difference (D), and Ratio (R) where the equity dimensions were wealth status, education level, sex of child, place of residence, and subnational regions (divisions). For each measure, point estimates along with a 95% confidence interval (CI) were reported.

**Results:**

An uprising pattern in the prevalence of early initiation of breastfeeding was found, where early initiation of breastfeeding increased from 24.9% to 2004 to 59.0% in 2017. We found significant wealth-driven inequalities in early initiation of breastfeeding practice in every wave of survey favoring the poorest wealth quintile (in 2017, D -10.5; 95% CI -16.6 to -4.3). We also identified geographical disparities in early initiation of breastfeeding practice (in 2017, PAF 11.1; 95% CI 2.2 to 19.9) favoring the Rangpur (65.5%), and Sylhet (65.3%) divisions. Education-related disparities were observed in 2004 only, but not in later survey years, which was due to a much lower level of adherence among those with secondary or higher education. There were no significant disparities in early initiation of breastfeeding based on the urban vs. rural residence and sex of the child.

**Conclusions:**

The highest attention should be placed in Bangladesh to attain the WHO’s 100% recommendation of timely initiation of breastfeeding. This study emphasizes on addressing the existing socioeconomic and geographic inequalities. Awareness-raising outreach programs focusing the mothers from wealthier sub-groups and divisions with lower prevalence should be planned and implemented by the joint effort of the government and non-government organizations.

## Background

According to the World Health Organization (WHO), breastfeeding should begin within the child’s first hour of life and continues as long as the mother and the child desire [1]. Early initiation of breastfeeding, equivalent to placing the newborn on the mother’s breast within the first hour of birth, is crucial for the newborn’s survival and growth and establishing breastfeeding practice over a longer period [2]. Early initiation of breastfeeding practice in South Asia is only 39%, which is the lowest among different regions around the globe [2]. Bangladesh, however, manifested better early initiation of breastfeeding practice ranging from 51.24 to 61.19% [3, 4] but falling short of the WHO’s 100% early initiation of breastfeeding recommendation [5]. Breastfeeding is the universally accepted single best infant feeding practice source, providing numerous benefits to the newborn and mother pair. Breastfeeding is advocated as a vital public health approach for decreasing newborn and child mortality, mother morbidity, and reduction of the cost of medical treatment for both maternal and childhood illnesses [6].

Despite significant benefits to the mother and the newborn, there is significant variability in early initiation of breastfeeding uptake between and within countries. There are disparities in early initiation of breastfeeding practice based on geographic region and other socioeconomic conditions. For instance, a recent study using BDHS 2017-18 data demonstrated that early initiation of breastfeeding is highly prevalent in Rangpur (66.57%) and Sylhet (66.13%) division due to its local norms and culture as well as the decreased cesarean delivery rates [4]. Numerous research has looked at the socioeconomic factors and other maternal and child-related factors that are associated with early initiation of breastfeeding in Bangladesh [7, 8], and in other countries [9–11]. Early initiation of breastfeeding practices were found to be more prevalent among women from rural areas than those from urban areas, who were less educated compared to higher educated [7]. In addition, factors such as age at first marriage and first birth [7], family wealth index, place of delivery [12, 13], mode of delivery [7, 14, 15], antenatal care visit [7], mothers’ BMI [7, 16] are prominently associated with early initiation of breastfeeding.

Even though a considerable decrease in newborn mortality over time, the Millennium Development Goal 4 (MDG-4) has not made enough progress [17]. Goal 3 of the Sustainable Development Goals (SDGs) focused on promoting MDG-4, which aims to achieve the SDG targets by lowering the maternal mortality rate (MMR) to 70 deaths per 100,000 live births, reducing under-five mortality to 25 per 1000 live births, and reducing newborn mortality to 12 per 1000 live births [18]. The sustainable targets for reducing newborn and maternal mortality would be impacted by resolving socioeconomic inequalities in early initiation of breastfeeding [19]. Moreover, it would be possible to construct more targeted and efficient treatments by thoroughly examining the disparity of early initiation of breastfeeding across various dispersion measurements [20].

Several studies were conducted in Bangladesh to identify the prevalence of early initiation of breastfeeding and its associated factors [14, 21]. To the best of our knowledge, only one study [22] looked at the disparity in early initiation of breastfeeding practice in Bangladesh using the BDHS 2017-18. However, no studies have been done to estimate the magnitude and especially the trend of inequalities in early initiation of breastfeeding in Bangladesh over time applying the WHO-recommended socioeconomic disparity trend. Therefore, this study aims to estimate the trends and magnitude of both socioeconomic and geographical inequalities of early initiation of breastfeeding practice in Bangladesh over the previous two decades using five BDHS data. The outcome of this research would be of interest to the government and the public health policymakers in Bangladesh to emphasize the program design focusing on specific equity-dimension to reduce the inequalities and thus fostering the 100% coverage of early initiation of breastfeeding recommended by WHO [5].

## Methods

### Study design and data source

The BDHS data from the survey period of 2004 to 2017 were used in this study. BDHS collects data on key demographics and health indicators including maternal and child health as a part of the DHS MEASURE program conducted in over 90 countries. In Bangladesh, this survey is conducted by the National Institute of Population Research and Training (NIPORT) and the Ministry of Health and Family Welfare of Bangladesh in collaboration with USAID. A two-stage stratified cluster sampling technique is used by the BDHS for the collection of survey data. In the first stage, the enumeration areas (EAs) were selected based on the previous national census in Bangladesh which were considered as the primary sampling units (PSUs) for the survey. Then, from each enumeration area, the households were selected in the second stage of sampling. The latest BDHS 2017-18 final report has a detailed description of the methodologies including the sampling techniques used in this survey [3]. We used data that is deposited in the Health Equity Assessment Toolkit (HEAT) of WHO.

### Outcome variable

Early initiation of breastfeeding was the outcome variable for this study. Data were collected from the mothers having at least one live birth two to three years preceding the survey [3]. Women who put their babies to breast within the first hour of birth for breastfeeding were considered to have early initiation of breastfeeding [23]. A binary response was created for the outcome variable and coded “1” if the breastfeeding was initiated within the first hour of birth and “0” if the initiation of breastfeeding was after 1st hour [3].

### Equity dimensions

Economic status, educational level, place of residence, sex of the child, and subnational regions are the five different dimensions on which inequalities were measured. The categories of the economic status (household wealth quintile) were poorest, poorer, middle, richer, and richest sub-groups. Household wealth quintile was constructed using the principal component analysis (PCA) considering the household income, assets, and other characteristics [24]. The educational level of the women was divided into three categories as no education, primary, and secondary or higher. Sex of the children was considered as male and female. The place of residence was divided into urban and rural and for the sub-national regions, the eight administrative divisions of Bangladesh were considered.

### Statistical analysis

The reproductive, maternal, newborn, and child health dataset from the WHO health inequality monitor data repository was analyzed using the HEAT software by WHO to measure the inequalities in early initiation of breastfeeding [25]. Out of the available inequality dimensions in the updated version of the software, we used five inequality dimensions in socioeconomic and geographic domains. Four summary measures; Difference (D), Population Attributable Risk (PAR), Population Attributable Fraction (PAF), and Ratio (R) were used to measure the inequalities across all the inequality dimensions. Both absolute and relative measures were used in this study since summary metrics from absolute and relative measures provide precise health inequalities according to WHO.

Among the summary measures, D is a simple as well as absolute measure, whereas R is a simple but relative measure. Both of the measures considered the highest and lowest subgroups for calculating inequality. On the other hand, PAR, and PAF are complex measures, where PAR is an absolute measure and PAF is a relative measure. For the favorable variable like breastfeeding the formula for calculation of D is as follows.

1 $${\rm{D = }}{{\rm{Y}}_{{\rm{highest}}}}{\rm{- }}{{\rm{Y}}_{{\rm{lowest}}}}$$

The difference is calculated between the highest and the lowest group as no reference group is defined. R is measured as the ratio of two subgroups from the following equation.

2 $${\rm{R = }}{{\rm{Y}}_{{\rm{high}}}}{\rm{/ }}{{\rm{Y}}_{{\rm{low}}}}$$

The complex measure PAF and PAR acknowledge the size of the population subgroup and require population average for calculation. For this study we considered the national average $\mu$ as the population average for the calculation of the complex measures. The PAR is obtained from the Eq. 3.

3 $${\rm{PAR = }}{{\rm{Y}}_{{\rm{ref}}}}-\mu$$

Here $\mu$ is the national average. The Y_ref_ for the ordered variable is the most advantageous group and for the non-ordered variable it is the subgroup with highest prevalence. The PAF is calculated from the PAR and the national average and multiplying the result by 100.

4 $${\rm{PAF}}\,{\rm{ = }}\,{\rm{}}\frac{{{\rm{PAR}}}}{{\rm{\mu }}}\,{\rm{ \times }}\,{\rm{100}}$$

For both ordered and non-ordered variable the PAF is calculated from the Eq. 4. The detailed calculation techniques of all the summary measures are described elsewhere [26, 27].

The larger value in any of the summary measures indicates higher inequality irrespective of the direction of the value. The positive value indicates the inequality in the early initiation of breastfeeding is concentrated toward the advantageous group and the negative value indicates the inequality is concentrated toward the disadvantaged subgroup in each dimension. Inequality will be absent if the D, PAF, and PAR values are 0. In contrast, inequality will be absent if the value of R becomes 1. To show the significance of the results, we calculated a 95% confidence interval (CI) for inequality measures along with their point estimates. The inequality measures D, PAF, and PAR were considered significant if the CIs did not include 0, and that for R was considered significant if the CI did not include 1.

## Results

### Distribution of early initiation of breastfeeding in different equity dimensions

**Fig. 1:**
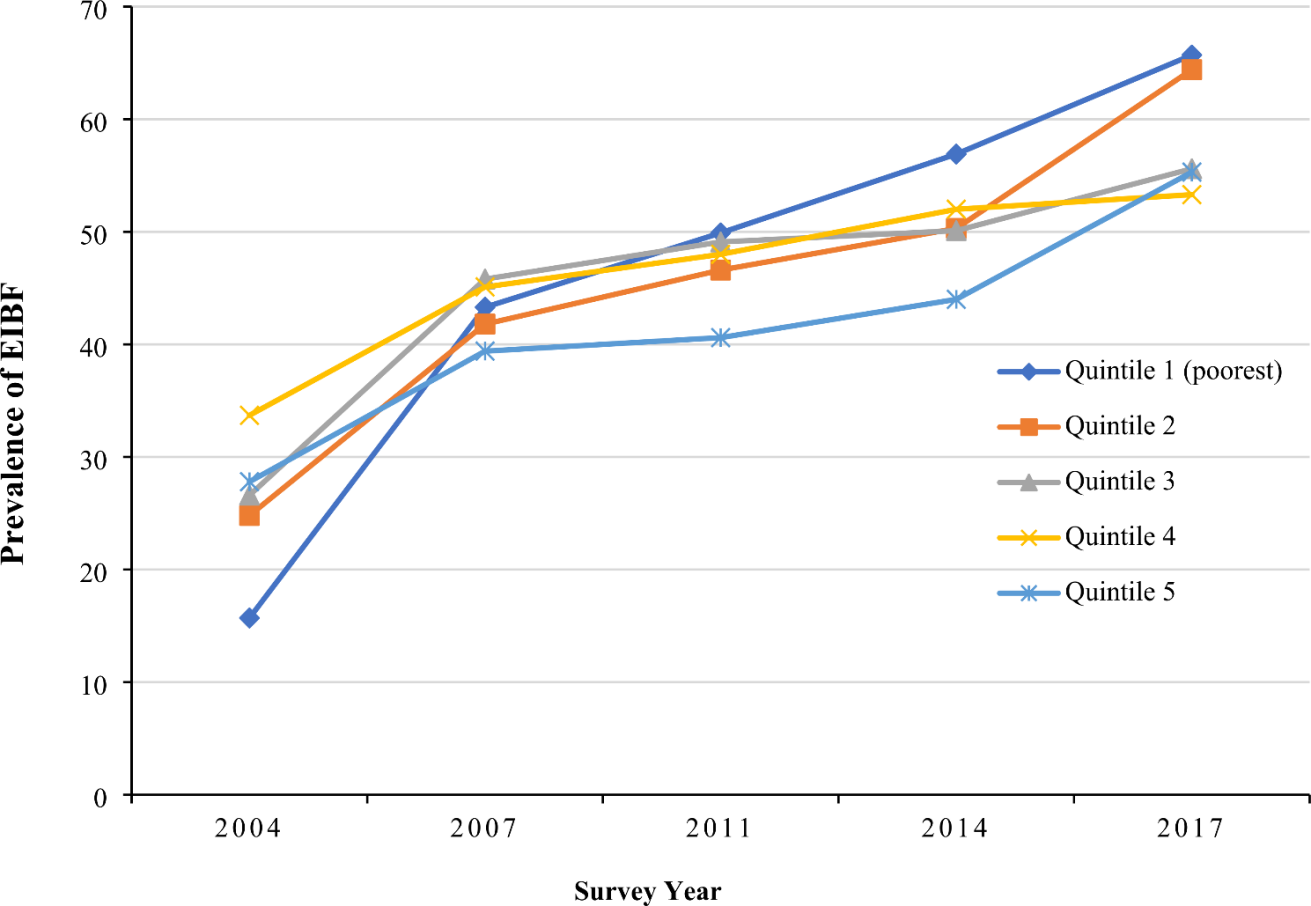
EIBF practice in Bangladesh based on different wealth quintiles: evidence from BDHS (2004–2017)

**Fig. 2:**
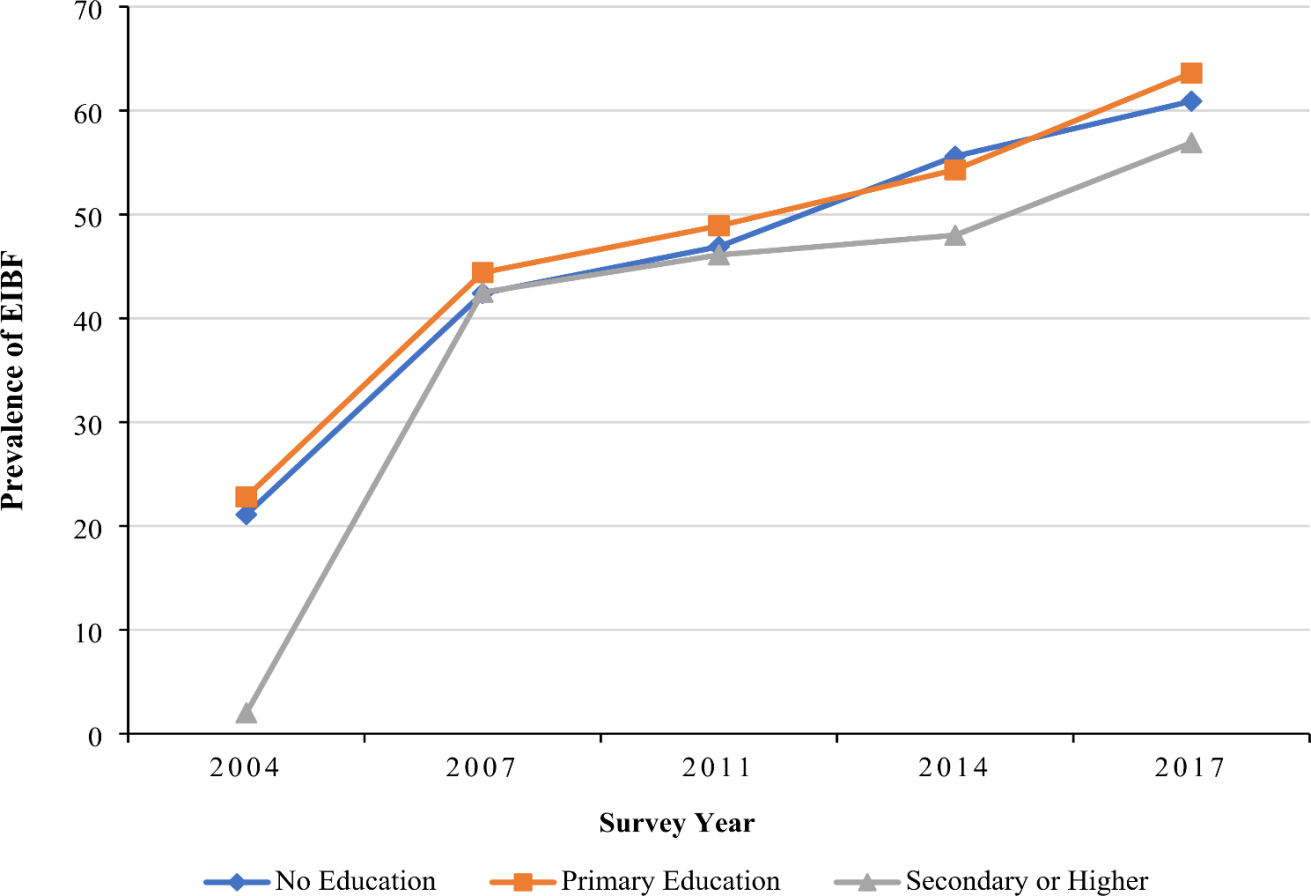
EIBF practice in Bangladesh segregated by the level of education: evidence from BDHS (2004–2017)

The national prevalence of early initiation of breastfeeding was found to have an increasing trend from 24.9% to 2004 to 59.0% in 2017. The trend of early initiation of breastfeeding practice from 2004 to 2017 among Bangladeshi women of different socioeconomic status is shown in Fig. 1. An uprising trend in early initiation of breastfeeding practice was observed among all sub-groups, while women from the richest quintile were found to have the highest early initiation of breastfeeding practice in 2004 (27.8%), however, in 2017, the highest prevalence was observed among those from the poorest wealth quintile (65.7%). Similarly, an increasing pattern of early initiation of breastfeeding practice across all education sub-groups was observed from 2004 to 2017 (Fig. 2).

**Fig. 3:**
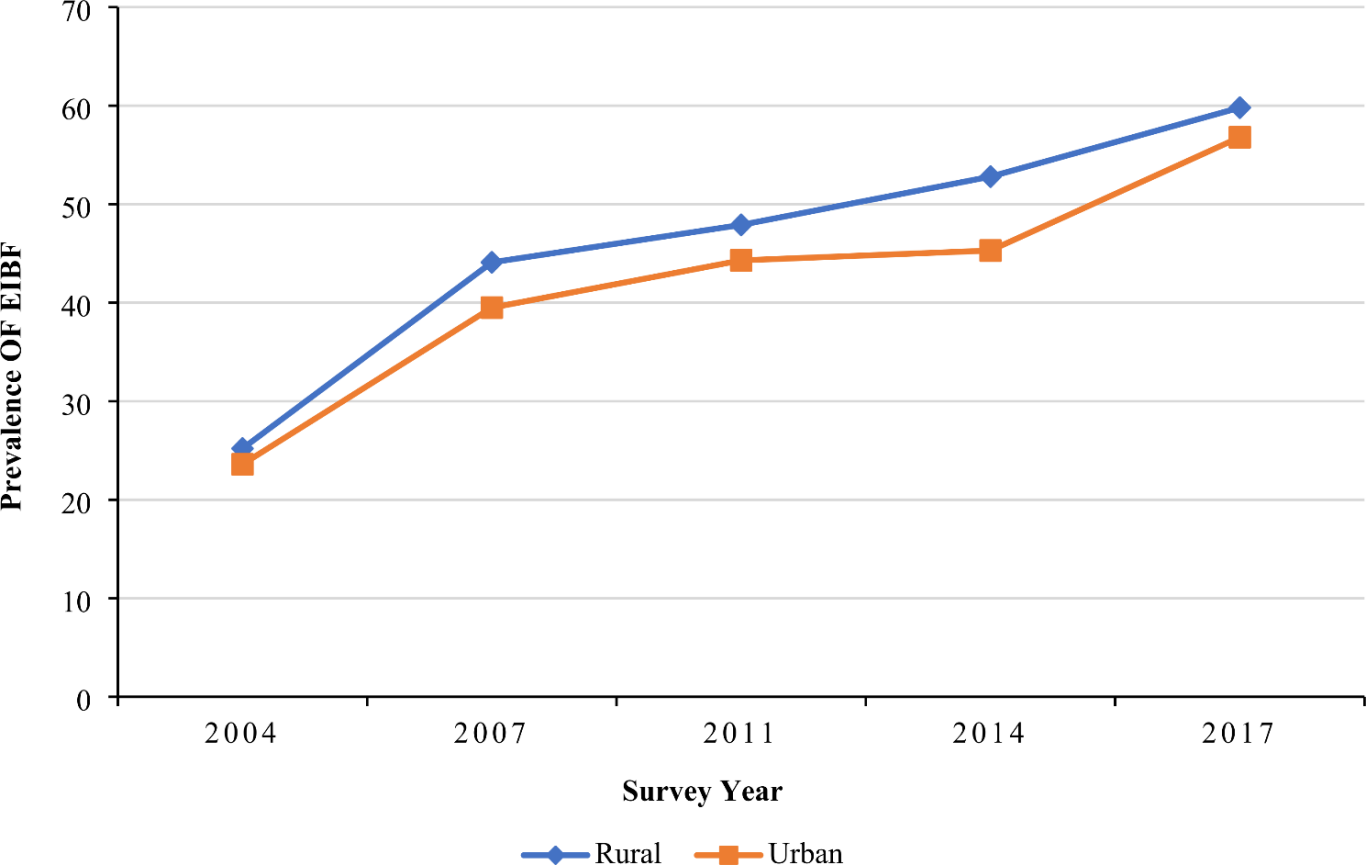
EIBF practice in Bangladesh segregated by the place of residence: evidence from BDHS (2004–2017)

**Fig. 4:**
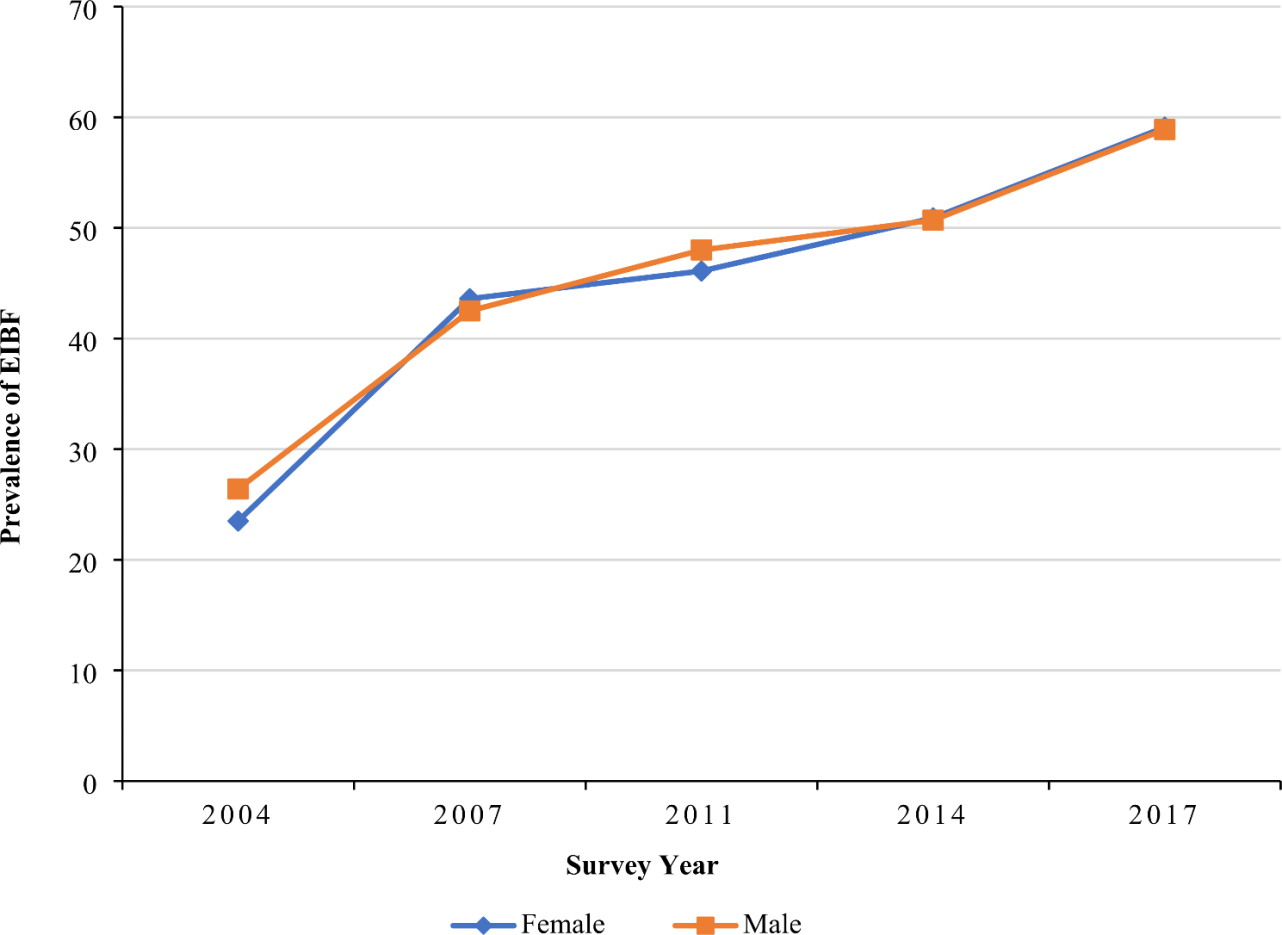
EIBF practice in Bangladesh segregated by the sex of child: evidence from BDHS (2004–2017)

Furthermore, women from rural areas (59.8%) had a higher prevalence of early initiation of breastfeeding than those living in urban areas (56.8%) in 2017 (Fig. 3). An increasing trend of early initiation of breastfeeding prevalence was observed among both male and female children from 2004 to 2017; however, both sub-groups showed almost similar prevalence in 2017 (59.1% in female, and 58.9% in male children) (Fig. 4). Women belonging to the Rangpur (65.5%) division had the highest prevalence of early initiation of breastfeeding in 2017 followed by the Sylhet division (65.3%), while participants from Khulna division had the lowest prevalence of early initiation of breastfeeding in the last two consecutive surveys (39.1% in 2014, and 49.2% in 2017) (Table 1).

**Table 1:** Trends in the prevalence of early initiation of breastfeeding, disintegrated across four inequality dimensions, from years 2004 to 2017

Inequality Dimension	2004 (24.9%)		2007 (43.1%)		2011 (47.1%)		2014 (50.8%)		2017 (59.0%)	
	n	Estimate (95% CI)	n	Estimate (95% CI)	n	Estimate (95% CI)	n	Estimate (95% CI)	n	Estimate (95% CI)
**Economic Status**										
Quintile 1 (poorest)	658	15.7 (13.0, 18.8)	472	43.3 (38.3, 48.5)	718	49.9 (45.1, 54.8)	699	56.9 (51.1, 62.5)	721	65.7 (61.6, 69.5)
Quintile 2	510	24.8 (20.7, 29.4)	504	41.8 (36.7, 47.0)	652	46.6 (42.0, 51.2)	618	50.3 (44.3, 56.2)	736	64.4 (60.5, 68.1)
Quintile 3	563	26.6 (22.2, 31.5)	443	45.8 (40.3, 51.4)	647	49.1 (44.5, 53.7)	641	50.1 (45.4, 54.7)	672	55.6 (50.9, 60.2)
Quintile 4	450	33.7 (28.2, 39.7)	447	45.1 (39.2, 51.1)	673	48.0 (43.9, 52.2)	631	52.0 (47, 56.9)	710	53.3 (49.2, 57.3)
Quintile 5 (richest)	434	27.8 (23.9, 32.2)	430	39.4 (34.5, 44.4)	576	40.6 (36.5, 44.9)	616	44.0 (39.4, 48.7)	658	55.2 (50.4, 59.9)
**Level of Education**										
No Education	891	21.1 (18.3, 24.3)	514	42.4 (36.9, 48.1)	551	46.9 (41.8, 52.1)	433	55.6 (48.7, 62.2)	222	60.9 (53.0, 68.3)
Primary School	809	22.8 (19.8, 26.0)	684	44.4 (40.2, 48.7)	971	48.9 (44.9, 53.0)	905	54.3 (48.3, 60.2)	969	63.6 (60.1, 67.0)
Secondary / Higher	916	30.5 (26.8, 34.4)	1089	42.5 (38.8, 46.3)	1742	46.1 (43.4, 48.8)	1867	48 (45.2, 50.8)	2307	56.9 (54.4, 59.3)
**Place of Residence**										
Rural	2118	25.2 (22.9, 27.7)	1796	44.1 (40.6, 47.6)	2526	47.9 (45.3, 50.5)	2370	52.8 (49.6, 55.9)	2569	59.8 (57.3, 62.3)
Urban	498	23.6 (19.9, 27.7)	499	39.5 (35.6, 43.5)	738	44.3 (40.7, 48.0)	836	45.3 (40.5, 50.1)	928	56.8 (52.9, 60.6)
**Sex of child**										
Female	1328	23.5 (21.1, 26.0)	1143	43.6 (39.8, 47.6)	1592	46.1 (43.0, 49.2)	1500	50.9 (47.5, 54.3)	1685	59.1 (56.2, 61.9)
Male	1288	26.4 (23.6, 29.5)	1152	42.5 (39.2, 45.9)	1673	48 (45.1, 50.9)	1705	50.7 (47.1, 54.2)	1812	58.9 (56.2, 61.5)
**Sub-National Regions**										
Barisal	157	20.9 (15.4, 27.7)	132	50.8 (45.5, 56.1)	177	43.6 (37.0, 50.3)	185	52.0 (45.0, 59.0)	209	60.3 (55.7, 64.8)
Chattogram	567	19.7 (15.1, 25.3)	526	34.9 (29.6, 40.5)	783	46.2 (41.5, 50.8)	682	45.8 (40.8, 50.8)	738	54.0 (48.3, 59.6)
Dhaka	794	23.5 (20.0, 27.5)	729	43.7 (38.9, 48.6)	988	43.0 (38.6, 47.5)	1182	51.8 (45.8, 57.8)	887	60.6 (55.5, 65.4)
Khulna	280	28.3 (24.4–32.6)	193	51.9 (42.6–61.1)	305	45.7 (40.5–50.9)	250	39.1 (34.0-44.5)	322	49.2 (42.9–55.6)
Mymensingh	-	-	-	-	-	-	-	-	298	64.0 (58.9, 68.8)
Rajshahi	595	29.2 (24.7, 34.2)	522	43.4 (35.9, 51.3)	439	53.5 (47.1, 59.8)	323	52.5 (47.5, 57.6)	387	57.6 (52.2, 62.9)
Rangpur	-	-	-	-	334	50.8 (44.7, 56.8)	292	59.9 (54.5, 65.0)	379	65.5 (60.1, 70.5)
Sylhet	224	30.0 (24.9, 35.8)	193	48.0 (41.6, 54.4)	238	54.0 (48.5, 59.4)	292	56.6 (50.1, 62.9)	278	65.3 (60.2, 70.0)

CI: Confidence Interval, Mymensingh division was separated from Dhaka division in 2015, and Rangpur division was separated from Rajshahi division in 2010. Hence, the estimates for BDHS 2004–2014 data of Mymensingh, and BDHS 2004–2007 data of Rangpur division are not shown in the table.

### Magnitude of the inequalities in early initiation of breastfeeding

In this study, we found significant wealth-driven inequality and geographical disparities in early initiation of breastfeeding in Bangladesh over time. Wealth-driven disparities were obtained by both absolute (D) and relative (R) measures with a higher concentration among the poorest sub-group from 2007 to 2017 compared to those from households with the richest wealth quintile. For instance, the absolute difference (D) of -10.5 (95% CI -16.6 to -4.3) in 2017 shows significant inequalities favoring those from the highest wealth quintile. However, no significant rural-urban inequality and sex disparity regarding early initiation of breastfeeding were identified. Regarding the educational level of women, significant inequality was observed in 2004, which didn’t show any significant inequalities in later survey waves. For instance, the relative PAF measure of 22.4 (95% CI 13.3 to 31.4) in 2004 shows significant inequalities favoring those having secondary or higher education compared to those having no formal education. We also found significant inequalities in sub-national regions from 2004 to 2017 using both simple (D, R) and complex measures (PAF, PAR). With a higher concentration of early initiation of breastfeeding practice in the Sylhet division in the last two consecutive surveys, the regional inequalities were demonstrated with a declining trend disfavoring the Khulna division. The significant regional inequalities were estimated from the PAF of 11.1 (95% CI 2.2 to 19.9) as the relative measure and PAR of 26.5 (95% CI 1.3 to 11.7) as the absolute measure in 2017 (Table 2).

**Table 2:** Inequality indices estimates of the factors associated with the prevalence of early initiation of breastfeeding, from years 2004–2017

Inequality Dimension	2004		2007		2011		2014		2017	
	Estimate	95% CI	Estimate	95% CI	Estimate	95% CI	Estimate	95% CI	Estimate	95% CI
**Economic status**										
D	12.2	7.1, 17.2	-3.9	-11.0, 3.1	-9.3	-15.7, -2.9	-12.9	-20.3, -5.6	-10.5	-16.6, -4.3
PAF	11.7	1.6, 21.9	0.0	-9.3, 9.3	0.0	-6.9, 6.9	0.0	-6.4, 6.4	0.0	-5.3, 5.3
PAR	2.9	0.4, 5.5	0.0	-4.0, 4.0	0.0	-3.2, 3.2	0.0	-3.3, 3.3	0.0	-3.1, 3.1
R	1.8	1.4, 2.3	0.9	0.8, 1.1	0.8	0.7, 0.9	0.8	0.7, 0.9	0.8	0.8, 0.9
**Level of Education**										
D	9.3	4.5, 14.1	0.1	-6.6, 6.8	-0.8	-6.7, 5	-7.6	-14.9, -0.3	-4.0	-12.1, 4.1
PAF	22.4	13.3, 31.4	0.0	-8.8, 8.8	0.0	-8.1, 8.1	0.0	-8.6, 8.6	0.0	-10.6, 10.6
PAR	5.6	3.3, 7.8	0.0	-3.8, 3.8	0.0	-3.8, 3.8	0.0	-4.4, 4.4	0.0	-6.2, 6.2
R	1.4	1.2, 1.7	1.0	0.9, 1.2	1.0	0.9, 1.1	0.9	0.8, 1.0	0.9	0.8, 1.1
**Place of Residence**										
D	-1.7	-6.2, 2.9	-4.6	-9.8, 0.7	-3.5	-8.1, 1.0	-7.5	-13.2, -1.8	-3.0	-7.6, 1.5
PAF	0.0	-3.2, 3.2	0.0	-2.5, 2.5	0.0	-2.0, 2.0	0.0	-2.0, 2.0	0.0	-1.7, 1.7
PAR	0.0	-0.8, 0.8	0.0	-1.1, 1.1	0.0	-0.9, 0.9	0.0	-1.0, 1.0	0.0	-1.0, 1.0
R	0.9	0.8, 1.1	0.9	0.8, 1.0	0.9	0.8, 1.0	0.9	0.8, 1.0	0.9	0.9, 1.0
**Sex of child**										
D	-3.0	-6.7, 0.8	1.1	-4.0, 6.2	-1.9	-6.2, 2.3	0.3	-4.7, 5.2	0.2	-3.7, 4.1
PAF	0.0	-6.8, 6.8	1.3	-3.4, 6.0	0.0	-3.6, 3.6	0.3	-2.9, 3.5	0.2	-2.5, 2.9
PAR	0.0	-1.7, 1.7	0.5	-1.5, 2.6	0.0	-1.7, 1.7	0.1	-1.5, 1.8	0.1	-1.5, 1.7
R	0.9	0.8, 1.0	1.0	0.9, 1.2	1.0	0.9, 1.0	1.0	0.9, 1.1	1.0	0.9, 1.1
**Sub-National Region**										
D	10.3	2.9, 17.8	17.0	6.3, 27.8	11.0	3.9, 18.0	20.7	13.3, 28.1	16.3	8.0, 24.5
PAF	20.6	8.7, 32.5	20.5	12.1, 28.9	14.8	9.3, 20.3	17.8	6.3, 29.4	11.1	2.2, 19.9
PAR	5.1	2.2, 8.1	8.8	5.2, 12.5	7.0	4.4, 9.5	9.1	3.2, 14.9	6.5	1.3, 11.7
R	1.5	1.1, 2.1	1.5	1.2, 1.9	1.3	1.1, 1.5	1.5	1.3, 1.8	1.3	1.1, 1.5

CI: Confidence Interval, D: Difference, PAR: Population Attributable risk, PAF: Population Attributable Fraction, R: Ratio.

## Discussion

This study aimed to measure the magnitude of early initiation of breastfeeding and respective inequalities in Bangladesh from the BDHS data over the last five surveys. We identified an increasing trend in the prevalence of early initiation of breastfeeding from 24.9% to 2004 to 59.0% in 2017. While looking at the equity dimensions, we found significant wealth-driven inequality and geographical disparities in early initiation of breastfeeding in Bangladesh over the years. However, no significant rural-urban inequality and sex disparity regarding early initiation of breastfeeding were identified. Regarding the educational level of women, significant inequality was found in 2004 but didn’t show any significant inequalities in later survey waves.

This study showed that the prevalence of early initiation of breastfeeding increased gradually from 2004 to 2017. A similar finding was manifested in the previous studies conducted in Bangladesh [21], Ethiopia [20], Ghana [28], and Indonesia [29]. Bangladesh entered the good state in early initiation of breastfeeding according to the WHO in 2014 transitioning from a poor state in 2004. But Bangladesh is far behind in achieving the WHO recommendation of an early initiation of breastfeeding prevalence of 100%. This might be due to the presence of an unequal increase in the prevalence of breastfeeding initiation among different subgroups [5]. The increasing trend in the initiation of breastfeeding within 1st hour of life could be due to the increase in the health-related knowledge of early initiation of breastfeeding, its importance in childhood development, and increased awareness among mothers [4]. Another plausible explanation can be the extensive effort of the government of Bangladesh and non-government organizations in creating awareness about the importance of early initiation of breastfeeding through advertisement in the mass media, and different campaigns providing counseling and community mobilization [30].

Inequality in early initiation of breastfeeding among the wealth quintile was found significant over the survey period where the poorest subgroup was interestingly found advantageous with higher early initiation of breastfeeding among the poor compared to the rich in all survey waves except in 2004. This finding was in line with the study conducted in Bangladesh [4] and Ethiopia [20] and in contradiction with several other studies [9, 15, 31]. The contradictory result could be simply due to the better awareness of Bangladeshi women about early initiation of breastfeeding irrespective of their wealth status [4]. It could also be explained by the fact that poor women mostly deliver at home by normal vaginal delivery, while delivery by cesarean-section is more prevalent among women from households with rich wealth status according to previous studies conducted in Bangladesh [7, 32, 33]. The BDHS reports and several previous literature also supports that women delivered at the hospital or via the cesarian section are less likely to initiate breastfeeding early [4, 15].

Educated-related inequality in early initiation of breastfeeding was found in 2004 survey point, and later it didn’t show any significant disparities. Previous study conducted in Bangladesh also didn’t find any significant association between maternal education and early initiation of breastfeeding [14]. The plausible explanation behind this finding could be due to the overall better awareness among the mothers in Bangladesh. Besides, a study that reviewed the country programs used to promote breastfeeding argued that the Government of Bangladesh didn’t adopt any national strategy to promote Infant and Young Child Feeding (IYCF) as part of the Global Strategy for IYCF until 2007 [23, 34]. Also, no significant inequalities were observed in the urban/rural or child sex dimensions. Contrasting results indicating significant differences in the prevalence of early initiation of breastfeeding based on maternal education in Ethiopia [35], and place of residence in Jordan [36] were found. The credible reason behind this could be due to the increase in the awareness campaign about breastfeeding including the dispersion of messages through mass media irrespective of place of residence, sex of child, and education of the parents [34].

Significant regional disparities in the early initiation of breastfeeding were observed in Rangpur having the highest prevalence of early initiation of breastfeeding, and Khulna having the lowest consecutively in the last two surveys. Significant regional disparities were also found in the studies conducted in Bangladesh [4, 7, 21] and Ethiopia [20]. The lower prevalence of early initiation of breastfeeding in Khulna could be due to the higher prevalence of cesarian section among the women and which may act as an important contributing factor [14]. Participants from the Sylhet division were found to have a higher prevalence of early initiation of breastfeeding than any other division. This might be due to the difference in the cultural norms of the community [7], access to the health facility, and health-related information [3, 15]. Besides the higher rate of home delivery, the lower rate of cesarian section and the higher number of skilled birth attendants working on early initiation of breastfeeding in Sylhet compared to other divisions could also play an important role in this disparity [3, 7, 15].

### Strengths and limitations

In this study, the trend and magnitude of inequalities in early initiation of breastfeeding have been measured based on five equity dimensions over the years from the last five rounds of BDHS data. The use of both simple and complex (considering the weight) measures to determine the inequality in both socioeconomic and geographic domains makes the result more appropriate and intuitive. The chosen analytical technique eventually strengthens the quality of the study.

Alongside the strength, our study has some limitations as well. Firstly, the use of data from the secondary source limits us from establishing any temporal relationship between the independent and outcome variable. Secondly, the retrospective nature of the data collection might make the study prone to recall bias. Thirdly, the result could not reflect the current scenario of early initiation of breastfeeding as the analysis was based on the time when data was generated. Lastly, the use of this specific analytic technique restricted us from considering other variables.

## Conclusions

In Bangladesh inequalities in the early initiation of breastfeeding were found consistently significant in economic and subnational regional dimensions. Although the inequality in the educational level, place of residence, and sex of child were not statistically significant, the fluctuating prevalence was observed in different survey years. Despite the improvement in all the dimensions over time, wealth-driven inequality in the early initiation of breastfeeding still exists and might significantly affect childhood development. The highest attention should be placed in Bangladesh to attain the WHO’s 100% recommendation of timely initiation of breastfeeding. This study emphasizes on addressing the existing socioeconomic and geographic inequalities. To minimize the inequality in the initiation of breastfeeding within the first hour of life and eventually ensure better early childhood development, both government and NGOs should plan and implement awareness-raising outreach programs keeping in mind different influential factors of early initiation of breastfeeding addressing especially the recently delivered and expecting mothers in the wealthier sub-group, and from divisions with lower early initiation of breastfeeding prevalence in Bangladesh.

## Data Availability

The study used data from the 2017–2018 Bangladesh Demographic and Health Survey. The data set is available at: https://dhsprogram.com/data/available-datasets.cfm.

## Abbreviations

BDHS: Bangladesh Demographic and Health Survey
EA: Enumeration Area
HEAT: Health Equity Assessment Toolkit
IYCF: Infant and Young Child Feeding
MDG: Millennium Development Goals
MMR: Maternal Mortality Rate
NGO: Non-Government Organization
PSU: Primary Sampling Unit; CI: Confidence Interval
PAF: Population Attributable Fraction
PAR: Population Attributable Risk
PCA: Principal Component Analysis
SDG: Sustainable Development Goals
WHO: World Health Organization
